# Effect of Workflow Improvements on Time to Endovascular Thrombectomy for Acute Ischemic Stroke in the MR CLEAN Registry

**DOI:** 10.1161/SVIN.122.000733

**Published:** 2023-04-28

**Authors:** Paula M. Janssen, Bob Roozenbeek, Jonathan M. Coutinho, Adriaan C.G.M. van Es, Wouter J. Schonewille, Geert J. Lycklama a Nijeholt, Hester F. Lingsma, Diederik W.J. Dippel, MR CLEAN Registry Investigators

**Affiliations:** ^1^ Department of Neurology Erasmus MC University Medical Center Rotterdam Rotterdam The Netherlands; ^2^ Department of Neurology Amsterdam UMC Amsterdam The Netherlands; ^3^ Department of Radiology Leiden University Medical Center Leiden The Netherlands; ^4^ Department of Neurology Sint Antonius Hospital Nieuwegein The Netherlands; ^5^ Department of Radiology The Hague Medisch Centrum Haaglanden The Netherlands; ^6^ Department of Public Health Erasmus MC University Medical Center Rotterdam Rotterdam The Netherlands

## Abstract

**Background:**

Insight in the effect of workflow improvements can help to minimize the time between onset of ischemic stroke and start of endovascular thrombectomy (EVT). The authors aimed to assess the implementation of EVT workflow strategies and their effect on time to treatment.

**Methods:**

The authors used data from the MR CLEAN (Multicenter Randomized Controlled Trial of Endovascular Treatment for Acute Ischemic Stroke in The Netherlands) registry and included patients with acute ischemic stroke in the anterior circulation, who underwent EVT between March 2014 and November 2017. Data on implementation of 20 predefined workflow improvement strategies during the study period were collected from each intervention center. Multilevel linear regression with a random intercept for center was used to quantify the effect of each strategy on door‐to‐groin puncture time, with adjustment for calendar time, for directly presented and transferred patients separately.

**Results:**

The authors included 2633 patients who were treated in 14 intervention centers. Of the 20 predefined strategies, 18 were actually implemented in ≥1 centers during the study period. In directly presented patients (n=1157), the intervention with the largest effect on door‐to‐groin puncture time was a strategy to avoid sedation during EVT compared with standard use of general anesthesia, which led to a reduction of 29% (95% CI, 6–46; *P*=0.02), corresponding to a decrease of 26 minutes (95% CI, 5–42). In transferred patients (n=1476), the interventions with the largest decrease in door‐to‐groin puncture time were a strategy to make the decision for patient transfer to the angiosuite after 1 stroke physician assessed the imaging, instead of both interventionist and neurologist (47% [95% CI, 5–70]; 19 minutes [95% CI, 2–29]) (*P*=0.03), and a strategy to perform neurological assessment at the angiosuite instead of the emergency department (32% [95% CI, 19–43]; 13 minutes [95% CI, 8–17]) (*P*<0.001).

**Conclusion:**

Intervention centers have implemented multiple new strategies to improve their workflow. Such workflow improvements lead to substantial reductions in time to EVT and may thereby improve the outcome of patients with acute ischemic stroke.

## Graphical abstract

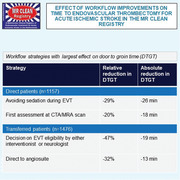

**Nonstandard Abbreviations and Acronyms**
DTGT: door‐to‐groin puncture time
EVT: endovascular thrombectomy
NIHSS: National Institutes of Health Stroke Scale
PERFEQTOS: Performance Feedback on the Quality of Care in Hospitals Performing Thrombectomy for Ischemic Stroke
PSC: primary stroke center
MR CLEAN: Multicenter Randomized Controlled Trial of Endovascular Treatment for Acute Ischemic Stroke in The Netherlands

**Clinical Perspective**

- Multiple strategies to effectively improve workflow and time to treatment in endovascular thrombectomy for acute ischemic stroke were identified.
- The type and number of implemented workflow improvement strategies over time varied largely between intervention centers.
- Intervention centers should critically evaluate their current workflow and determine their own opportunities to shorten time to endovascular thrombectomy and thereby improve outcome of patients with acute ischemic stroke.

Endovascular thrombectomy (EVT) has been proven effective in select patients with acute ischemic stroke caused by large vessel occlusion in the anterior circulation.^1^, ^2^, ^3^ A shorter time to treatment leads to a more favorable outcome.^4^, ^5^ Acute stroke care is therefore being reorganized to optimize the time from stroke onset to EVT in order to improve patient outcome, as is recommended by current international guidelines.^6^, ^7^ Possible targets for the improvement of EVT workflow can be found in both the prehospital phase and the in‐hospital phase. Acute stroke care systems, consisting of emergency medical services, primary stroke centers (PSCs), and intervention centers, are being challenged to identify these targets, and to create and implement strategies for workflow improvements within their own system.

A systematic review of studies on workflow improvements in EVT identified mainly observational and quasi‐experimental pre–post intervention studies, performed in single centers and with small sample sizes.^8^ Included studies analyzed the effects of workflow improvements concerning prehospital management, in‐hospital patient transfer, anesthetic management, teamwork, and feedback. Although heterogeneity was considerable between studies, a meta‐analysis suggested that workflow improvements are associated with a significant reduction in time to treatment. However, given the aforementioned limitations of the included studies and the potential publication bias, a multicenter, prospective study of unselected centers with consecutive patient inclusion is needed to identify effective interventions to improve workflow.

Despite considerable advances in acute stroke care, many stroke care systems still struggle to implement EVT in their workflow.^9^ Increasing the insight in the implementation of EVT in daily practice and in the effect of EVT workflow improvements on time to treatment may help to further optimize acute stroke care. We aimed to assess the implementation of EVT workflow improvement strategies and their effect on time to treatment.

## Methods

### Workflow Improvement Strategies

Based on literature review^8^ and expert consensus, we selected 20 workflow improvement strategies for our study (Table 1). We invited principal investigators of all 19 intervention centers that included patients in the MR CLEAN (Multicenter Randomized Controlled Trial of Endovascular Treatment for Acute Ischemic Stroke in The Netherlands) registry in December 2018 to complete an online survey regarding the implementation of the 20 selected workflow improvement strategies in their centers between March 2014 and November 2017. Centers could indicate whether each workflow improvement strategy had been implemented or changed during the study period, and specify the date of implementation or change (Table S1 in Supplement).

**Table 1:** Workflow Improvement Strategies for EVT.

Workflow improvement strategy—short term	Full description of the strategy; with possible answers in the online survey
Prenotification	Prenotification of patients with suspected acute stroke to the intervention center by the general practitioner, EMS, or referring neurologist from a PSC; yes or no
Vascular imaging at PSC	Performance of vascular imaging (CTA or MRA) at the PSCs; yes or no
Cloud‐based image sharing	Cloud‐based image sharing between PSCs and the intervention center; yes or no
Activation interventional team—direct patients	Moment of activation of the interventional team in patients presenting directly to the intervention center; at prenotification, after clinical assessment, or after imaging
Activation interventional team—transferred patients	Moment of activation of the interventional team in patients who have been transferred from a PSC; at prenotification from the PSC to the intervention center, after clinical assessment in the intervention center, or after imaging in the intervention center
Assessing physician	Physician who performs the first clinical assessment of patients with acute stroke who might be eligible for EVT; from the department of neurology, from the department of neurointervention, from the ED
Location of first assessment—direct patients	Location of first clinical assessment of patients with acute stroke presenting directly to the intervention center; at the ED without CTA or MRA available in the same room, in a room with CTA or MRA available, or at the angiosuite
Location of first assessment—transferred patients	Location of first clinical assessment of patients with acute stroke who have been transferred from a PSC; at the ED without CTA or MRA available in the same room, in a room with CTA or MRA available, or at the angiosuite
No‐turn‐back approach	Patients are transferred within the intervention center only to locations that they have not been before; for example, a patient is not transferred from the ED to the CT scan and back to the ED again; yes or no
Combined vascular imaging and treatment	Initial vascular imaging (CTA, MRA, or digital subtraction angiography) is performed in the same room as where the EVT is performed; yes or no
Decision‐making	Moment of decision‐making on eligibility for EVT, followed by transfer of the patient to the angiosuite and completion of the preparations for the EVT procedure at the angiosuite; after both the interventionist and the neurologist have personally assessed the imaging, or after either the interventionist or the neurologist has assessed the imaging
Anesthetic management	Standard anesthetic management during EVT; no sedation or pain medication, local anesthesia, conscious sedation, or general anesthesia
EVT material set	A standard material set for EVT for acute ischemic stroke is available for use at the angiosuite within 5 minutes; yes or no
Bladder catheter	A bladder catheter is placed before EVT in every patient; yes or no
Real‐time visualization timeline	A smartphone app or (digital) system is available for real‐time visualization of the current timeline of a patient who might be eligible for EVT; yes or no
Regular meetings with EMS	Regular meetings are organized at least 3 times a year with the EMS to discuss care and outcome of patients with acute stroke receiving EVT; yes or no
Regular meetings with ED	Regular meetings are organized at least 3 times a year with the ED to discuss care and outcome of patients with acute stroke receiving EVT; yes or no
Regular meetings with interventional team	Regular meetings are organized at least once a month between neurologists and the interventional team to discuss care and outcome of individual patients with acute stroke receiving EVT; yes or no
Regular meetings with PSCs	Regular meetings are organized at least 3 times a year with the referring neurologist from PSCs to discuss care and outcome of patients with acute stroke receiving EVT; yes or no
Written protocol	Individual tasks of all staff involved in EVT for acute ischemic stroke are recorded in ≥1 written protocols. Staff involved are staff members from EMS, ED, and the departments of neurology, neurointervention, or anesthesiology; yes or no

CT indicates computed tomography; CTA, computed tomography angiography; ED, emergency department; EMS, emergency medical services; EVT, endovascular thrombectomy; MRA, magnetic resonance angiography; and PSC, primary stroke center.

### Study Population

We used data from the MR CLEAN registry, a multicenter, prospective cohort study, which started directly after the final MR CLEAN trial randomization in March 2014, and includes all consecutive patients with acute ischemic stroke undergoing EVT in The Netherlands. The MR CLEAN registry has previously been described in more detail.^10^ A central medical ethics committee evaluated the study protocol of the MR CLEAN registry and granted permission to perform the study as a registry, as the study required no additional interventions or procedures beyond those performed as usual care. Data from the MR CLEAN registry cannot be made available for purposes of reproducing the results or replicating the procedure, as no patient approval was obtained for sharing coded data. Syntax and output files of statistical analyses will be made available upon reasonable request.

For the present study, we only included patients who were treated with EVT between March 2014 and November 2017 in an intervention center that had completed our online survey.

Other inclusion criteria were large vessel occlusion in the anterior circulation, age 18 years and older, and EVT that started within 6.5 hours after start of symptoms or last seen well.

### Data Collection and Outcomes

We collected data on patient characteristics, including age, sex, previous ischemic stroke, diabetes, atrial fibrillation, hypertension, hypercholesterolemia, smoking status, prestroke modified Rankin scale score, baseline National Institutes of Health Stroke Scale (NIHSS) score, occlusion side, and occlusion segment on computed tomography angiography. We also collected data on workflow, concerning treatment with intravenous alteplase, transfer status (first presentation at a PSC or at an intervention center), time from onset of stroke to reperfusion, time from onset of stroke to arrival at the intervention center (“door”), time from door‐to‐groin puncture time (DTGT), and time from groin puncture to reperfusion. Onset of stroke was defined as the time of symptom onset or the time of last seen well. Time of reperfusion was defined as the time of successful reperfusion, last contrast bolus, or end of procedure. Collected data on patient outcome were modified Rankin scale score at 90 days and NIHSS score at 24 to 48 hours. Primary outcome was DTGT.

### Statistical Analysis

First, we described the frequency of implementation, and changes in implementation, of the 20 workflow improvement strategies for EVT.

Second, we used an interrupted time series design to evaluate the effect of the improvement strategies. Our study includes different intervention centers implementing strategies at different time points. This design allows to account for independent time trends as well as between‐center differences other than the intervention of interest. We used regression models to estimate the effect of each improvement strategy on the DTGT. We excluded strategies that were applied in <20 patients, and strategies that were applied uniformly in all intervention centers. Each patient was categorized for each workflow strategy, depending on the patients’ date of EVT and the date of implementation of the strategy, or change in strategy in the center in which the patient was treated. To take into account improvement over time independent of the implemented improvement strategies, we adjusted for calendar time. This was defined as the time from start of the measurement period (which differed between intervention centers) and EVT (continuous variable, in days, starting at 1). To capture other differences between centers, we used a random intercept for center in our regression models. DTGT was log transformed to approach a normal distribution. We used the formula (exp(coefficient)−1)*100 to calculate the percent change in DTGT for each strategy. To interpret the effect of the workflow strategies on DTGT, we also expressed the effect of each strategy as an increase or decrease in median DTGT in minutes. For this, we calculated the median DTGT for transferred and directly presented patients separately. We performed the analysis separately for transferred (patients with first presentation at a PSC) and directly presented patients (presenting directly to an intervention center), because we assumed that workflow and the effect of workflow interventions differ between these patients. Depending on the target of the workflow improvement strategy, only patients with a specific transfer status were included in the analysis. For example, to analyze the effect of the strategy concerning performance of vascular imaging at the PSC, only transferred patients were included.

In order to get unbiased effect estimates of regression effects, we substituted missing data using multiple imputation with 5 iterations, assuming missingness at random.^11^, ^12^ Descriptive baseline data are reported without imputation, and percentage missingness per variable is reported. We used Stata/SE statistical package version 16.0 (StataCorp LLC) for all analyses.

We report our results in accordance with the STROBE (Strengthening the Reporting of Observational Studies in Epidemiology) statement.^13^

## Results

The MR CLEAN registry enrolled 3637 patients treated in 19 centers between March 2014 and November 2017 (Figure S1 in Supplement). A total of 3357 patients met our inclusion criteria regarding age, location of stroke, and time from onset to start of EVT. Our online survey regarding practice on EVT during the study period was completed by 14 intervention centers (74%) (Table S2 in Supplement). After exclusion of 724 patients who were treated in a nonresponding center, 2633 patients were included in our analysis.

### Baseline Characteristics

The mean age of the patients was 70 years (SD, 14 years) and 1380 patients (52%) were men (Table 2). Fifty‐eight percent (n=1475) of patients were transferred from a PSC to an intervention center. One center only treated patients who presented directly to that center. The DTGT decreased over time (Figure S2 in Supplement). In patients who presented directly to the intervention center, median DTGT decreased from 112 minutes (interquartile range [IQR], 90–140 minutes) in 2014 to 82 minutes (IQR, 65–108 minutes) in 2017. Median DTGT for transferred patients decreased from 67 minutes (IQR, 49–90 minutes) in 2014 to 35 minutes (25–49 minutes) in 2017.

**Table 2:** Baseline Patient Characteristics

	MR CLEAN registry n=2633; 14 centers	Missing values, n (%)
Age, mean (SD), y	70 (14)	0
Men, n (%)	1380 (52)	0
Medical history, n (%)		
Previous stroke	424 (17)	85 (3.2)
Diabetes	391 (15)	83 (3.1)
Atrial fibrillation	614 (24)	96 (3.6)
Hypercholesterolemia	743 (30)	172 (6.5)
Hypertension	1329 (53)	111 (4.2)
Smoking	554 (29)	709 (27)
Prestroke mRS score, n (%)		116 (4.4)
0	1704 (68)	
1	339 (14)	
2	193 (7.7)	
>2	281 (11)	
NIHSS score at baseline, median (IQR)	16 (11–19)	116 (4.4)
Occlusion side, left hemisphere, n (%)	1410 (54)	34 (1.3)
Occlusion segment, n (%)		291 (11)
ICA	612 (26)	
M1	1372 (59)	
M2	343 (15)	
Other (M3, ACA)	15 (0.6)	
Intravenous alteplase treatment, n (%)	1939 (76)	71 (2.6)
Transfer from PSC to intervention center, n (%)	1475 (58)	68 (2.6)
Time from stroke onset to reperfusion in min, median (IQR)	255 (202–315)	244 (9.3)
Time from stroke onset to door intervention center in min, median (IQR)	137 (68–190)	201 (7.6)
Time from door intervention center to groin puncture in min, median (IQR)	57 (35–87)	308 (12)
Time from groin puncture to reperfusion in min, median (IQR)	49 (30–74)	286 (11)
mRS score at 90 days, median (IQR)	3 (2–6)	289 (11)
NIHSS score at 24–48 hours	10 (4–17)	374 (14)

ACA indicates anterior cerebral artery; ICA, internal carotid artery; IQR, interquartile range; M1, middle cerebral artery segment 1; M2, middle cerebral artery segment 2; M3, middle cerebral artery segment 3; MR CLEAN, Multicenter Randomized Controlled Trial of Endovascular Treatment for Acute Ischemic Stroke in The Netherlands; mRS, modified Rankin scale; NIHSS, National Institutes of Health Stroke Scale; and PSC, primary stroke center.

### Implementation of Workflow Strategies

**Figure 1:**
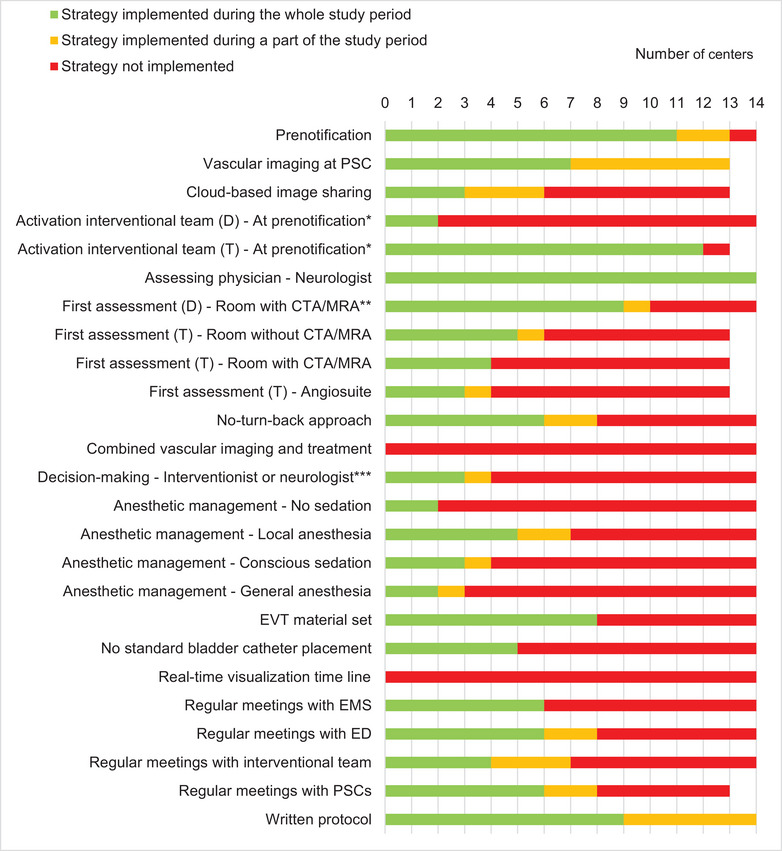
**Implementation of workflow improvement strategies for endovascular thrombectomy (EVT) in 14 intervention centers**. * Compared with “after imaging,” ** compared with “without computed tomography angiography (CTA)/magnetic resonance angiography (MRA),” and *** compared with “both interventionist and neurologist.” One center only treated patients who presented directly to that center; therefore, the total number of centers is 13 for strategies concerning only transferred patients. D indicates patients presenting directly at the intervention center; and T, transferred patients.

Of the 20 predefined workflow improvement strategies, 1 strategy was uniformly applied in all centers and 2 were not applied in any of the centers (Figure 1). Twelve workflow improvement strategies changed in ≥1 intervention centers during the study period (Table S3 in Supplement). Strategies that were implemented in the largest number of centers were the performance of vascular imaging at the PSC (6 centers) and the availability of a written protocol describing individual tasks of all staff involved in EVT (5 centers). Two intervention centers changed 5 strategies. Four centers did not change any of the investigated workflow strategies during the study period.

### Effect of Workflow Strategies on Time to Treatment

**Figure 2:**
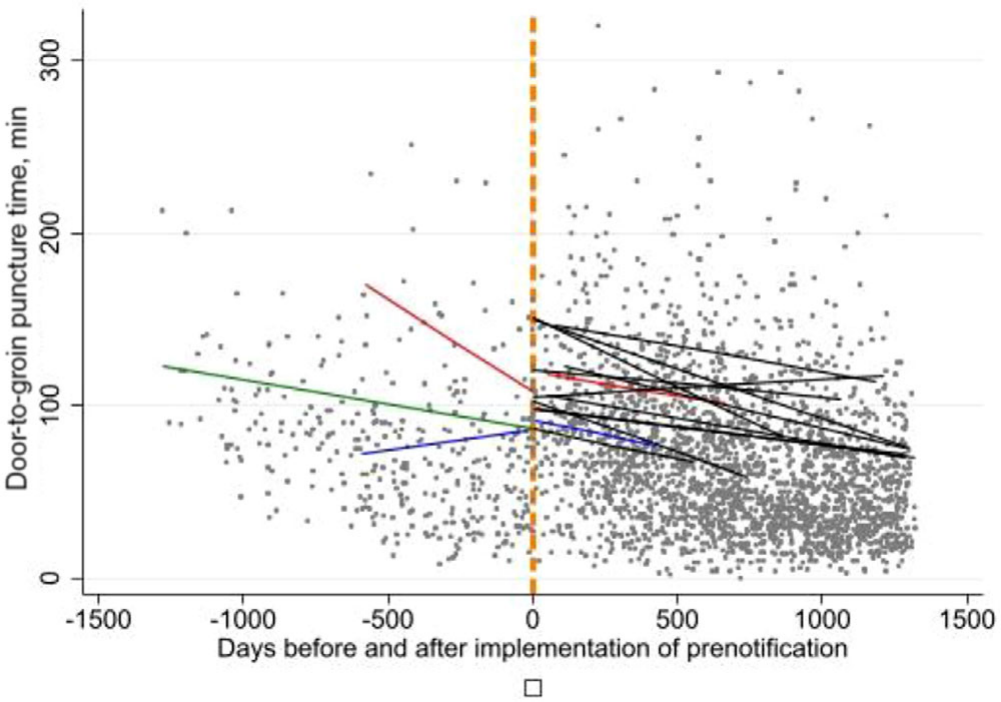
**Interrupted time series of the workflow improvement strategy concerning prenotification in directly presented patients**. This figure shows the effect of implementation of prenotification in each intervention center on door‐to‐groin puncture time (DTGT). Each dot represents 1 patient. The vertical orange dashed line represents the moment of implementation of prenotification. One intervention center (green line) did not apply prenotification during the study period (between March 2014 and November 2017). Two intervention centers (red and blue lines) started without prenotification and changed this strategy during the study period. Eleven hospitals (black lines) applied prenotification during the whole study period.

For patients presenting directly to an intervention center (n=1157), the effect of 12 workflow improvement strategies was analyzed (Table 3). In these patients, the strategy concerning standard anesthetic management during EVT had the largest impact on DTGT. Directly presented patients who were treated in an intervention center in which the standard procedure at that time was to avoid sedation during EVT had a shorter DTGT time compared with directly presented patients treated in an intervention center, in which applying general anesthesia was the standard procedure at that time. DTGT decreased by 29% (95% CI, 6–46; *P*=0.02), corresponding to a decrease of 26 minutes (95% CI, 5–42). Other workflow improvement strategies with a significant effect on the DTGT in directly presented patients were the performance of the first clinical assessment of patients with acute stroke at computed tomography or magnetic resonance imaging scanner (decrease of 20% [95% CI, 8–31]; 18 minutes [95% CI, 7–27]) (*P*=0.002) and the application of local anesthesia during EVT compared with general anesthesia (decrease of 17% [95% CI, 2–31]; 16 minutes [95% CI, 1–28]) (*P*=0.03). As an example, the interrupted time series of the strategy concerning prenotification is visualized in Figure 2.

**Table 3:** Effect of Workflow Improvement Strategies on DTGT in Patients Presenting Directly to the Intervention Center for Endovascular Thrombectomy.

Strategy	Number of hospitals (no. of patients) in intervention/control group	Change in DTGT (95% CI), %	Estimated change in DTGT, (95% CI), min	*P* value
Prenotification	13 (1005)/3 (152)	15 (−0.4 to 33)	14 (−0.3 to 30)	0.06
Activation interventional team: at prenotification, compared with after imaging	2 (196)/12 (961)	−8 (−33 to 26)	−7 (−30 to 23)	0.59
Location of first assessment: room with CTA/MRA scanner compared with room without	10 (795)/5 (362)	−20 (−31 to −8)	−18 (−27 to −7)	0.002
No‐turn‐back approach	8 (584)/8 (573)	−6 (−18 to 8)	−6 (−17 to 7)	0.38
Decision‐making on eligibility, followed by transfer to angiosuite: after imaging assessment by either interventionist or neurologist, compared with both	4 (324)/11 (833)	12 (−16 to 21)	11 (−14 to 19)	0.90
Standard anesthetic management: no sedation compared with general anesthesia	2 (140)/3 (216)	−29 (−46 to −6)	−26 (−42 to −5)	0.02
Standard anesthetic management: local anesthesia compared with general anesthesia	7 (629)/3 (216)	−17 (−31 to −2)	−16 (−28 to −1)	0.03
Standard anesthetic management: conscious sedation compared with general anesthesia	4 (172)/3 (216)	−7 (−24 to 14)	−6 (−22 to 13)	0.49
EVT material set available for use within 5 min	8 (605)/6 (552)	8 (−14 to 35)	7 (−12 to 32)	0.49
No standard bladder catheter placement	5 (492)/9 (665)	0.8 (−20 to 28)	0.8 (−18 to 25)	0.94
Regular meetings with EMS	6 (437)/8 (720)	−5 (−24 to 20)	−4 (−22 to 18)	0.67
Regular meetings with ED	8 (506)/8 (651)	9 (−3 to 23)	8 (−3 to 20)	0.14
Regular meetings with interventional team	7 (392)/10 (765)	−3 (−2 to 10)	−3 (−2 to 9)	0.62
Written protocol	14 (944)/5 (213)	−4 (−13 to 5)	−4 (−12 to 5)	0.36

CTA indicates computed tomography angiography; DTGT, door‐to‐groin puncture time; ED, emergency department; EMS, emergency medical services; EVT, endovascular thrombectomy; and MRA, magnetic resonance angiography.

For transferred patients (n=1476), the effect of 15 workflow improvement strategies was analyzed (Table 4). The effect of the strategy concerning the moment of activation of the interventional team could not be analyzed due to a too small number of patients in the control group. For transferred patients, making the decision for patient transfer to the angiosuite for EVT after 1 stroke physician assessed the imaging, instead of both interventionist and neurologist, decreased DTGT the most, namely by 47% (95% CI, 5–70; 19 minutes [95% CI, 2–29]) (*P*=0.03). Neurological assessment of transferred patients at the angiosuite instead of the emergency department decreased the DTGT by 32% (95% CI, 19–43; 13 minutes [95% CI, 8–17]) (*P*<0.001). Other strategies that significantly improved the DTGT were the performance of vascular imaging at the PSCs (decrease of 15% [95% CI, 4–25]; 6 minutes [95% CI, 2–10]) (*P*=0.008) and the availability of cloud‐based image sharing between the PSCs and the intervention center (decrease of 19% [95% CI, 9–28]; 8 minutes [95% CI, 3–11]) (*P*=0.001).

**Table 4:** Effect of Workflow Improvement Strategies on DTGT Time in Patients Transferred to the Intervention Center for Endovascular Thrombectomy

Strategy	Number of hospitals (no. of patients) in intervention/control group	Change in DTGT (95% CI), %	Estimated change in DTGT (95% CI), min	*P* value
Prenotification	11 (1282)/3 (194)	3 (−16 to 26)	1 (−6 to 10)	0.79
Vascular imaging at PSC	12 (1217)/6 (259)	−15 (−25 to −4)	−6 (−10 to −2)	0.008
Cloud‐based image sharing	6 (792)/10 (684)	−19 (−28 to −9)	−8 (−11 to −3)	0.001
Location of first assessment: room with CTA/MRA scanner compared with room without	4 (443)/6 (649)	10 (−100 to 78)	4 (−40 to 31)	0.69
Location of first assessment: angiosuite compared with room without CTA/MRA scanner	4 (384)/6 (649)	−32 (−43 to −19)	−13 (−17 to −8)	<0.001
No‐turn‐back approach	7 (996)/8 (480)	−1 (−17 to 17)	−0.6 (−7 to 7)	0.87
Decision‐making on eligibility, followed by transfer to angiosuite: after imaging assessment by either interventionist or neurologist, compared with both	3 (319)/10 (1157)	−47 (−70 to −5)	−19 (−29 to −2)	0.03
Standard anesthetic management: no sedation compared with general anesthesia	2 (26)/3 (344)	−29 (−64 to 42)	−11 (−26 to 17)	0.36
Standard anesthetic management: local anesthesia compared with general anesthesia	7 (958)/3 (344)	−8 (−27 to 16)	−3 (−11 to 6)	0.48
Standard anesthetic management: conscious sedation compared with general anesthesia	3 (148)/3 (344)	4 (−19 to 33)	2 (−7 to 13)	0.74
EVT material set available for use within 5 min	7 (593)/6 (883)	27 (−25 to 115)	11 (−10 to 46)	0.38
No standard bladder catheter placement	5 (727)/8 (749)	−14 (−51 to 49)	−6 (−20 to 20)	0.58
Regular meetings with EMS	6 (573)/7 (903)	4 (−39 to 80)	2 (−16 to 32)	0.88
Regular meetings with ED	7 (836)/8 (640)	−3 (−17 to 13)	−1 (−7 to 5)	0.67
Regular meetings with interventional team	6 (659)/9 (817)	22 (5 to 41)	9 (2 to 16)	0.01
Regular meetings with PSCs	7 (681)/8 (795)	4 (−12 to 24)	2 (−5 to 10)	0.63
Written protocol	12 (1372)/5 (104)	−6 (−20 to 10)	−3 (−8 to 4)	0.42

CTA indicates computed tomography angiography; DTGT, door‐to‐groin puncture time; ED, emergency department; EMS, emergency medical services; EVT, endovascular thrombectomy; MRA, magnetic resonance angiography; and PSC, primary stroke center.

## Discussion

We studied the implementation of workflow improvement strategies for EVT in patients with acute ischemic stroke in daily practice. Our study shows that the majority of intervention centers has implemented ≥1 workflow improvements over time. This study also demonstrates that the type and number of implemented EVT workflow improvement strategies varies largely between intervention centers. We identified multiple workflow strategies that had a positive effect on time to treatment.

Only a quarter of the investigated workflow strategies were implemented in all intervention centers at the end of the study period. Variation in EVT workflow across hospitals and countries was also demonstrated in a survey among neurointerventionists in 2019.^14^ This variation is sometimes caused by structural factors, eg, whether the computed tomography scanner is located at the emergency department. Since our study and previous studies have shown that first assessment of a patient with acute stroke at the computed tomography or magnetic resonance imaging scanner,^15^ or at the angiosuite (transferred patients),^16^, ^17^, ^18^ can save valuable time, this factor should be taken into account when hospitals are designed or renovated.

In daily practice, many stroke care systems use the hub‐and‐spoke model in which patients with acute stroke eligible for EVT are transferred from PSCs to the interventions center. This eligibility for EVT is assessed using both patient and imaging characteristics. Our observation that a cloud‐based system of image sharing between PSCs and intervention center decreases time to treatment was also made by an observational study including 1 intervention center and 14 PSCs.^17^ The advantage of cloud‐based image sharing is that imaging can be assessed by the interventionalist before arrival of the patient, which not only saves time but also improves the preparation for the EVT itself.

Patients who presented directly to intervention centers in which no sedation or local anesthesia was the standard anesthetic procedure during EVT at that time had a shorter DTGT than patients treated in intervention centers in which general anesthesia was the standard procedure at that time. This possible benefit on DTGT of avoiding general anesthesia could not be demonstrated for transferred patients. Interpretation of these results concerning anesthetic management is complex. The impact of a shorter available time for preparation of the anesthetic support during EVT in directly presented patients compared with transferred patients is uncertain. Randomized controlled trials comparing general anesthesia with conscious sedation showed no difference in clinical outcomes between groups.^19^, ^20^, ^21^, ^22^, ^23^, ^24^, ^25^, ^26^ However, whether these trial results are also applicable in daily practice is uncertain, since each trial was performed in a single center and had a relatively small sample size. Furthermore, the effects of anesthetic management may also be influenced by the experience of the anesthesiologist in anesthetic support during EVT for acute ischemic stroke, and by the availability of an anesthesiologist. Nevertheless, treatment delay at the angiosuite should be minimized by a close collaboration of interventionist and anesthesiologist during EVT, and by having a local standard operating procedure for anesthetic management during EVT that is evaluated regularly.

Our survey shows that almost half of the intervention centers did not have regular meetings with each of the team members involved in the EVT workflow, such as staff from the emergency medical services, the emergency department, the interventional team, and the referring PSCs. Although our study could not demonstrate a direct effect on time to treatment, regular meetings in which both logistics as patient outcomes are evaluated can have a beneficial impact.^15^, ^27^, ^28^, ^29^, ^30^, ^31^ We encourage EVT teams, often led by stroke neurologists and interventionalists, to start and continue regular evaluations of EVT workflow with all involved team members.

We were not able to analyze the EVT workflow in all intervention centers in The Netherlands. However, considering the observed characteristics of both patients and centers, our study does provide an overview of EVT workflow in The Netherlands. Although some structural differences may exist compared with other regions or countries across the world, eg, concerning population density, results of our study may serve as inspiration to optimize workflow in every intervention center. Through critically evaluating current workflow using the improvement strategies of this study, each center can determine its own opportunities for improvement. This can also be supplemented by comparing workflow and onset to groin puncture times with other centers. This is being evaluated and implemented as an online dashboard in the Dutch PERFEQTOS (Performance Feedback on the Quality of Care in Hospitals Performing Thrombectomy for Ischemic Stroke) trial.^32^ Results of our study implicate that EVT workflow has not been optimized yet in everyday practice. This was also seen in a study including 195 Get With the Guidelines‐Stroke hospitals.^9^ Intervention centers should make efforts to further improve local workflow in order to optimize patient outcomes. An analysis of individual patient data from 5 EVT trials showed that for every 4‐minute delay in the time from door intervention center to reperfusion, 1 of every 1000 treated patients had a worse disability outcome.^5^

This study has several limitations. We analyzed the effect of workflow improvements by combining the date of EVT from each individual patient with the date of implementation of a workflow improvement in the intervention center. However, workflow improvements are not implemented perfectly in 1 day, or even in a month. If we assume a lag time, the effect of an improvement could be underestimated. Also, asking intervention centers to provide the date of changes in EVT workflow in retrospect could have led to recall bias. Moreover, in our study, there may have been deliberate deviations from the standard local workflow in individual patients, which we could not take into account in the analysis. This also occurs in daily practice, so our estimates do represent the actual effect in clinical practice. Nevertheless, we recommend each center to have a local standard, optimized, workflow. Furthermore, determining the effect of individual workflow improvements is more difficult when ≥2 workflow changes took place in the same period. These limiting factors may also explain why some workflow improvement strategies seemed to delay treatment in our study, eg, prenotification. This finding is opposite to previous studies, which have shown a positive effect of notification of the acute stroke team before arrival of the patient on time to treatment.^15^, ^27^, ^30^, ^33^, ^34^ Furthermore, we used data from 2014 to 2017. Although several workflow improvements may already have been implemented as standard care in some intervention centers, intervention centers that have started more recently to provide EVT or aim at further improving their workflow can benefit from our results. Since 2017, new workflow improvements have been developed, concerning, for example, availability of perfusion imaging and emergency medical services triage for large vessel occlusion. These factors were not included in our analysis, but reflect the necessity and possibility for continuous improvement in acute stroke care.

In conclusion, intervention centers have implemented multiple new strategies to improve their EVT workflow. Such workflow improvements lead to substantial reductions in time to treatment, and may thereby improve outcomes of patients with acute ischemic stroke.

## Sources of Funding

The MR CLEAN registry was partly funded by the TWIN Foundation, Erasmus MC University Medical Center Rotterdam, Maastricht University Medical Center, and Amsterdam UMC.

## Disclosures

Paula M. Janssen, Bob Roozenbeek, Wouter J. Schonewille, Geert J. Lycklama a Nijeholt, Adriaan C.G.M. van Es, and Hester F. Lingsma report no disclosures. Jonathan M. Coutinho has received grants paid to his institution from Boehringer Ingelheim, Bayer, and Medtronic. Diederik W.J. Dippel reports funding from the Dutch Heart Foundation, Brain Foundation Netherlands, The Netherlands Organisation for Health Research and Development, Health Holland Top Sector Life Sciences & Health, and unrestricted grants from Penumbra Inc., Stryker, Medtronic, Thrombolytic Science, LLC, and Cerenovus for research, all paid to the institution.

## Supporting information

Supplementary Information

## COLLABORATORS WHO NEED TO BE INDEXED IN PubMed

### MR CLEAN Registry Investigators – Group Authors

Aad van der Lugt, Charles B.L.M. Majoie, Yvo B.W.E.M. Roos, Robert J. van Oostenbrugge, Wim H. van Zwam, Jelis Boiten, Jan Albert Vos, Ivo G.H. Jansen, Maxim J.H.L. Mulder, Robert‐Jan B. Goldhoorn, Kars C.J. Compagne, Manon Kappelhof, Josje Brouwer, Sanne J. den Hartog, Wouter H. Hinsenveld, Bart J. Emmer, Wouter J. Schonewille, Marieke J.H. Wermer, Marianne A.A. van Walderveen, Julie Staals, Jeannette Hofmeijer, Jasper M. Martens, Geert J. Lycklama à Nijeholt, Sebastiaan F. de Bruijn, Lukas C. van Dijk, H. Bart van der Worp, Rob H. Lo, Ewoud J. van Dijk, Hieronymus D. Boogaarts, J. de Vries, Paul L.M. de Kort, Julia van Tuijl, Jo P. Peluso, Puck Fransen, Jan S.P. van den Berg, Boudewijn A.A.M. van Hasselt, Leo A.M. Aerden, René J. Dallinga, Maarten Uyttenboogaart, Omid Eschgi, Reinoud P.H. Bokkers, Tobien H.C.M.L. Schreuder, Roel J.J. Heijboer, Koos Keizer, Lonneke S.F. Yo, Heleen M. den Hertog, Emiel J.C. Sturm, Paul J.A.M. Brouwers, Marieke E.S. Sprengers, Sjoerd F.M. Jenniskens, René van den Berg, Albert J. Yoo, Ludo F.M. Beenen, Alida A. Postma, Stefan D. Roosendaal, Bas F.W. van der Kallen, Ido R. van den Wijngaard, Joost Bot, Pieter‐Jan van Doormaal, Anton Meijer, Elyas Ghariq, Reinoud P.H. Bokkers, Marc P. van Proosdij, G. Menno Krietemeijer, Dick Gerrits, Wouter Dinkelaar, Auke P.A. Appelman, Bas Hammer, Sjoert Pegge, Anouk van der Hoorn, Saman Vinke, H. Zwenneke Flach, Naziha el Ghannouti, Martin Sterrenberg, Wilma Pellikaan, Rita Sprengers, Marjan Elfrink, Michelle Simons, Marjolein Vossers, Joke de Meris, Tamara Vermeulen, Annet Geerlings, Gina van Vemde, Tiny Simons, Gert Messchendorp, Nynke Nicolaij, Hester Bongenaar, Karin Bodde, Sandra Kleijn, Jasmijn Lodico, Hanneke Droste, Maureen Wollaert, Sabrina Verheesen, D. Jeurrissen, Erna Bos, Yvonne Drabbe, Michelle Sandiman, Nicoline Aaldering, Berber Zweedijk, Jocova Vervoort, Eva Ponjee, Sharon Romviel, Karin Kanselaar, Denn Barning, Esmee Venema, Vicky Chalos, Ralph R. Geuskens, Tim van Straaten, Saliha Ergezen, Roger R.M. Harmsma, Daan Muijres, Anouk de Jong, Olvert A. Berkhemer, Anna M.M. Boers, J. Huguet, P.F.C. Groot, Marieke A. Mens, Katinka R. van Kranendonk, Kilian M. Treurniet, Manon L. Tolhuisen, Heitor Alves, Annick J. Weterings, Eleonora L.F. Kirkels, Eva J.H.F. Voogd, Lieve M. Schupp, Sabine L. Collette, Adrien E.D. Groot, Natalie E. LeCouffe, Praneeta R. Konduri, Haryadi Prasetya, Nerea Arrarte‐Terreros, and Lucas A. Ramos.
