# Supplementary material for: Effect of Workflow Improvements on Time to Endovascular Thrombectomy for Acute Ischemic Stroke in the MR CLEAN Registry
Source: Stroke Vasc Interv Neurol. 2023 Apr 28;3(4):e000733. doi: 10.1161/SVIN.122.000733 (PMC12778671; doi:10.1161/SVIN.122.000733)
Supplement: Supplementary file 1 — Supplementary Information [file SVI2-3-e000733-s001.pdf]

SUPPLEMENTAL MATERIALS

Effect of workflow improvements on time to endovascular thrombectomy for acute ischemic stroke in the MR CLEAN Registry

Paula M Janssen, Bob Roozenbeek, Jonathan M. Coutinho, Adriaan C.G.M. van Es, Wouter J Schonewille, Geert J Lycklama a Nijeholt, Hester F Lingsma, Diederik WJ Dippel, on behalf of the MR CLEAN Registry Investigators.

Affiliations

Erasmus MC University Medical Center Rotterdam, Department of Neurology (PJ, BR, DD) and Department of Public Health (HL), Rotterdam, The Netherlands.

Amsterdam UMC, Department of Neurology, Amsterdam, The Netherlands (JC).

Leiden University Medical Center, Department of Radiology, Leiden, The Netherlands (AvE).

Sint Antonius Hospital, Department of Neurology, Nieuwegein, The Netherlands (WS).

Medisch Centrum Haaglanden, Department of Radiology The Hague, The Netherlands (GL).

Table S1. Example questions from online survey among the principal investigators of interventions centers that included patients in the MR CLEAN Registry

Table S2. Characteristics of included and excluded MR CLEAN Registry intervention centers

Table S3. Changes in workflow for endovascular thrombectomy

Figure S1. Inclusion flowchart

Figure S2. Time trend of door-to-groin puncture time of stroke patients treated with endovascular thrombectomy between March 2014 and November 2017

MR CLEAN Registry Investigators – group authors

STROBE Statement - Checklist of items that should be included in reports of *cohort studies*

Table S1. Example questions from online survey among the principal investigators of interventions centers that included patients in the MR CLEAN Registry

**Question**	**Answer options**
Location of first assessment – direct patients	
Which of the following was standard procedure in your intervention center during the study period?<br><br>Stroke patients who present directly at the intervention center, are initially assessed at …	1. A room at the emergency department without CTA or MRA in that same room (entire study period)<br>2. A room where a CTA or MRA can be performed, which can be located at the emergency department or elsewhere at the hospital (entire study period)<br>3. The angiosuite (entire study period)<br>4. During the study period this location changed
When the answer was “4”, additional questions were asked	
During a part of the study period, was the initial assessment location “A room at the emergency department without CTA or MRA in that same room” ?	Yes or no<br><br>When “yes”, specify start and end date.
During a part of the study period, was the initial assessment location “A room where a CTA or MRA can be performed, which can be located at the emergency department or elsewhere”?	Yes or no<br><br>When “yes”, specify start and end date.
During a part of the study period, was the initial assessment location “The angiosuite”?	Yes or no<br><br>When “yes”, specify start and end date.

Table S2. Characteristics of included and excluded MR CLEAN Registry intervention centers

	**MR CLEAN Registry intervention centers included in our analysis (n=14)**	**MR CLEAN Registry intervention centers excluded from our analysis (n=5)**
Age in years, mean (SD)	70 (14)	70 (14)
NIHSS score at baseline, median (IQR)	11 (16-19)	11 (16-20)
Pre-stroke mRS score = 0 (%)	68	67
Time from door intervention center to groin puncture in minutes, median (IQR)	57 (35-87)	(67 (41-101)
Number of academic teaching hospitals (%)	5 (36)	2 (40)
Mean number of performed EVT in study period (range)	188 (23-398)	144 (79-210)

Table S3. Changes in workflow for endovascular thrombectomy

Workflow improvement strategies	Intervention Centers									
	A	B	C	D	E	F	G	I	J	K
Pre-notification	01-2016	09-2016								
Vascular imaging at PSC	01-2016		01-2015	01-2015	05-2017	11-2015	01-2016			
Cloud-based image sharing			07-2015			11-2016	01-2017			
Location of first assessment – direct patients								01-2017		
Location of first assessment – transferred patients			04-2015							
No-turn-back approach		09-2017							07-2015	
Decision-making	01-2016									
Anesthetic management			06-2016						01-2015	
Regular meetings with ED		01-2017				11-2016				
Regular meetings with interventional team	06-2017								07-2017	09-2017
Regular meetings with PSCs		05-2016					01-2015			
Written protocol	01-2017	05-2017				11-2015	01-2015	06-2017		

ED, emergency department; PSC, primary stroke center.

Ten centers, labeled A to K, changed one or more workflow improvement strategies during the study period (March 2014 to November 2017). Date (month-year) of implementation or change in strategy during the study period of each strategy is shown per center.

Figure S1. Inclusion flowchart

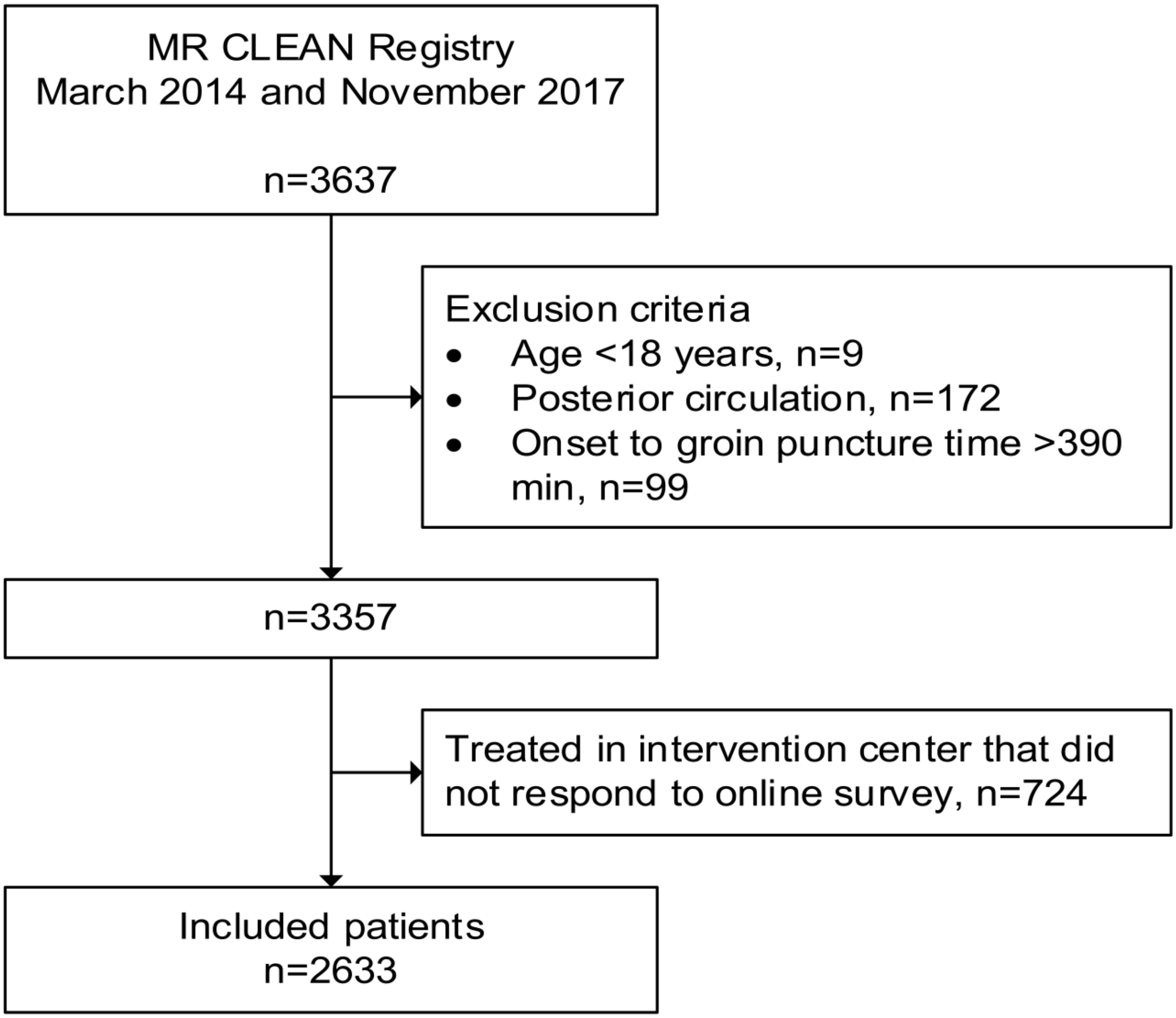

Figure S2. Time trend of door-to-groin puncture time of stroke patients treated with endovascular thrombectomy between March 2014 and November 2017

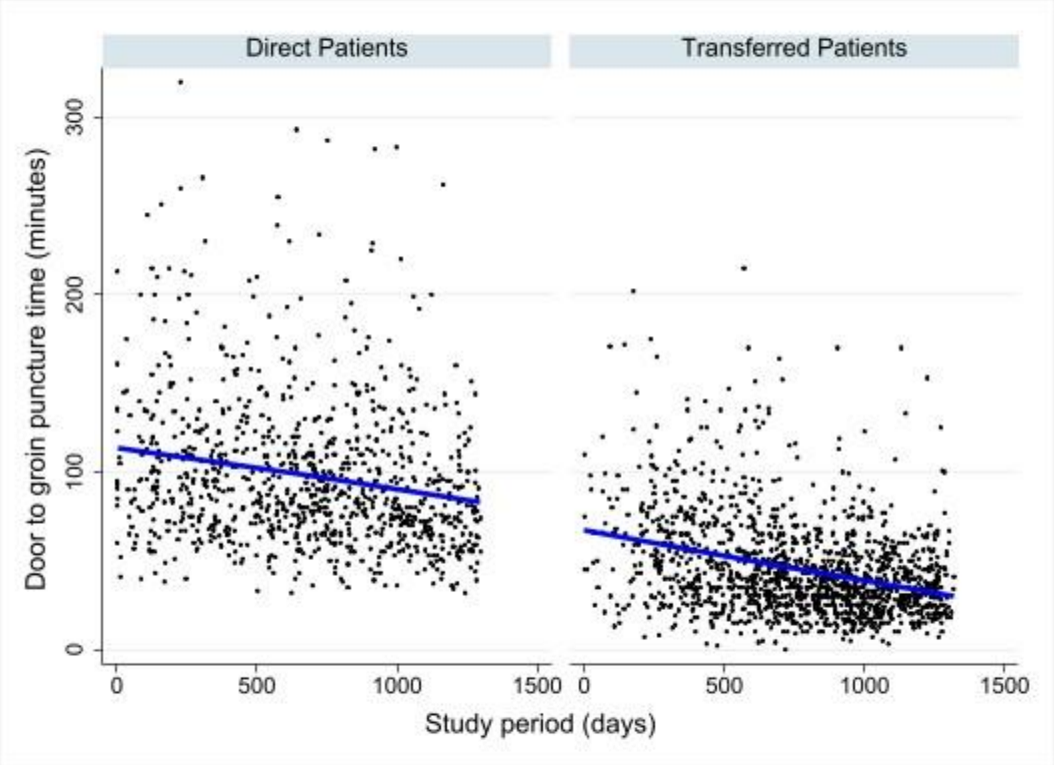

MR CLEAN Registry Investigators – group authors

Executive committee: Diederik W.J. Dippel[1];Aad van der Lugt[2];Charles B.L.M. Majoie[3];Yvo B.W.E.M. Roos[4];Robert J. van Oostenbrugge[5];Wim H. van Zwam[6];Jelis Boiten[14];Jan Albert Vos[8]

Study coordinators: Ivo G.H. Jansen[3];Maxim J.H.L. Mulder[1,2];Robert- Jan B. Goldhoorn[5,6];Kars C.J. Compagne[2];Manon Kappelhof[3];Josje Brouwer[4];Sanne J. den Hartog[1,2,40];Wouter H. Hinsenveld [5,6];
Local principal investigators: Diederik W.J. Dippel[1];Bob Roozenbeek[1];Aad van der Lugt[2];Adriaan C.G.M. van Es[2];Charles B.L.M. Majoie[3];Yvo B.W.E.M. Roos[4];Bart J. Emmer[3];Jonathan M. Coutinho[4];Wouter J. Schonewille[7];Jan Albert Vos[8]; Marieke J.H. Wermer[9];Marianne A.A. van Walderveen[10];Julie Staals[5];Robert J. van Oostenbrugge[5];Wim H. van Zwam[6];Jeannette Hofmeijer[11];Jasper M. Martens[12];Geert J. Lycklama à Nijeholt[13];Jelis Boiten[14];Sebastiaan F. de Bruijn[15];Lukas C. van Dijk[16];H. Bart van der Worp[17];Rob H. Lo[18];Ewoud J. van Dijk[19];Hieronymus D. Boogaarts[20];J. de Vries[22];Paul L.M. de Kort[21]; Julia van Tuijl[21] ; Jo P. Peluso[26];Puck Fransen[22];Jan S.P. van den Berg[22];Boudewijn A.A.M. van Hasselt[23];Leo A.M. Aerden[24];René J. Dallinga[25];Maarten Uyttenboogaart[28];Omid Eschgi[29];Reinoud P.H. Bokkers[29];Tobien H.C.M.L. Schreuder[30];Roel J.J. Heijboer[31];Koos Keizer[32];Lonneke S.F. Yo[33];Heleen M. den Hertog[22];Emiel J.C. Sturm[35]; Paul J.A.M. Brouwers[34]

Imaging assessment committee: Charles B.L.M. Majoie[3](chair);Wim H. van Zwam[6];Aad van der Lugt[2];Geert J. Lycklama à Nijeholt[13];Marianne A.A. van Walderveen[10];Marieke E.S. Sprengers[3];Sjoerd F.M. Jenniskens[27];René van den Berg[3];Albert J. Yoo[38];Ludo F.M. Beenen[3];Alida A. Postma[6];Stefan D. Roosendaal[3];Bas F.W. van der Kallen[13];Ido R. van den Wijngaard[13];Adriaan C.G.M. van Es[2];Bart J. Emmer[,3];Jasper M. Martens[12]; Lonneke S.F. Yo[33];Jan Albert Vos[8]; Joost Bot[36], Pieter-Jan van Doormaal[2]; Anton Meijer[27];Elyas Ghariq[13]; Reinoud P.H. Bokkers[29];Marc P. van Proosdij[37];G. Menno Krietemeijer[33];Jo P. Peluso[26];Hieronymus D. Boogaarts[20];Rob Lo[18];Dick Gerrits[35];Wouter Dinkelaar[2]Auke P.A. Appelman[29];Bas Hammer[16];Sjoert Pegge[27];Anouk van der Hoorn[29];Saman Vinke[20].

Writing committee: Diederik W.J. Dippel[1](chair);Aad van der Lugt[2];Charles B.L.M. Majoie[3];Yvo B.W.E.M. Roos[4];Robert J. van Oostenbrugge[5];Wim H. van Zwam[6];Geert J. Lycklama à Nijeholt[13];Jelis Boiten[14];Jan Albert Vos[8];Wouter J. Schonewille[7];Jeannette Hofmeijer[11];Jasper M. Martens[12];H. Bart van der Worp[17];Rob H. Lo[18]

Adverse event committee: Robert J. van Oostenbrugge[5](chair);Jeannette Hofmeijer[11];H. Zwenneke Flach[23]

Trial methodologist: Hester F. Lingsma[40]

Research nurses / local trial coordinators: Naziha el Ghannouti[1];Martin Sterrenberg[1];Wilma Pellikaan[7];Rita Sprengers[4];Marjan Elfrink[11];Michelle Simons[11];Marjolein Vossers[12];Joke de Meris[14];Tamara Vermeulen[14];Annet Geerlings[19];Gina van Vemde[22];Tiny Simons[30];Gert Messchendorp[28];Nynke Nicolaij[28];Hester Bongenaar[32];Karin Bodde[24];Sandra Kleijn[34];Jasmijn Lodico[34]; Hanneke

Droste[34];Maureen Wollaert[5];Sabrina Verheesen[5];D. Jeurrissen[5];Erna Bos[9];Yvonne Drabbe[15];Michelle Sandiman[15];Nicoline Aaldering[11];Berber Zweedijk[17];Jocova Vervoort[21];Eva Ponjee[22];Sharon Romviel[19];Karin Kanselaar[19];Denn Barning[10].

PhD / Medical students: Esmee Venema[40]; Vicky Chalos[1,40]; Ralph R. Geuskens[3]; Tim van Straaten[19];Saliha Ergezen[1]; Roger R.M. Harmsma[1]; Daan Muijres[1]; Anouk de Jong[1];Olvert A. Berkhemer[1,3,6];Anna M.M. Boers[3,39]; J. Huguet[3];P.F.C. Groot[3];Marieke A. Mens[3];Katinka R. van Kranendonk[3];Kilian M. Treurniet[3];Manon L. Tolhuisen[3,39];Heitor Alves[3];Annick J. Weterings[3],Eleonora L.F. Kirkels[3],Eva J.H.F. Voogd[11];Lieve M. Schupp[3];Sabine L. Collette[28,29];Adrien E.D. Groot[4];Natalie E. LeCouffe[4];Praneeta R. Konduri[39];Haryadi Prasetya[39];Nerea Arrarte-Terreros[39];Lucas A. Ramos[39].

List of affiliations MR CLEAN Registry Investigators – group authors

Department of Neurology[1], Radiology[2], Public Health[40], Erasmus MC University Medical Center;

Department of Radiology and Nuclear Medicine[3], Neurology[4], Biomedical Engineering & Physics[39], Amsterdam UMC, University of Amsterdam, Amsterdam;

Department of Neurology[5], Radiology[6], Maastricht University Medical Center and Cardiovascular Research Institute Maastricht (CARIM);

Department of Neurology[7], Radiology[8], Sint Antonius Hospital, Nieuwegein;

Department of Neurology[9], Radiology[10], Leiden University Medical Center;

Department of Neurology[11], Radiology[12], Rijnstate Hospital, Arnhem;

Department of Radiology[13], Neurology[14], Haaglanden MC, the Hague;

Department of Neurology[15], Radiology[16], HAGA Hospital, the Hague;

Department of Neurology[17], Radiology[18], University Medical Center Utrecht;

Department of Neurology[19], Neurosurgery[20], Radiology[27], Radboud University Medical Center, Nijmegen;

Department of Neurology[21], Radiology[26], Elisabeth-TweeSteden ziekenhuis, Tilburg;

Department of Neurology[22], Radiology[23], Isala Klinieken, Zwolle;

Department of Neurology[24], Radiology[25], Reinier de Graaf Gasthuis, Delft;

Department of Neurology[28], Radiology[29], University Medical Center Groningen;

Department of Neurology[30], Radiology[31], Atrium Medical Center, Heerlen;

Department of Neurology[32], Radiology[33], Catharina Hospital, Eindhoven;

Department of Neurology[34], Radiology[35], Medical Spectrum Twente, Enschede;

Department of Radiology[36], Amsterdam UMC, Vrije Universiteit van Amsterdam, Amsterdam;

Department of Radiology[37], Noordwest Ziekenhuisgroep, Alkmaar;

Department of Radiology[38], Texas Stroke Institute, Texas, United States of America.

STROBE Statement - Checklist of items that should be included in reports of *cohort studies*

	Item No	Recommendation	Page no.
**Title and abstract**	1	(*a*) Indicate the study's design with a commonly used term in the title or the abstract	1,3
		(*b*) Provide in the abstract an informative and balanced summary of what was done and what was found	3-4
**Introduction**			
Background/rationale	2	Explain the scientific background and rationale for the investigation being reported	5
Objectives	3	State specific objectives, including any prespecified hypotheses	5
**Methods**			
Study design	4	Present key elements of study design early in the paper	6,16
Setting	5	Describe the setting, locations, and relevant dates, including periods of recruitment, exposure, follow-up, and data collection	6-7
Participants	6	(*a*) Give the eligibility criteria, and the sources and methods of selection of participants. Describe methods of follow-up	6-7
		(*b*) For matched studies, give matching criteria and number of exposed and unexposed	NA
Variables	7	Clearly define all outcomes, exposures, predictors, potential confounders, and effect modifiers. Give diagnostic criteria, if applicable	7
Data sources/ measurement	8*	For each variable of interest, give sources of data and details of methods of assessment (measurement). Describe comparability of assessment methods if there is more than one group	7
Bias	9	Describe any efforts to address potential sources of bias	7-8
Study size	10	Explain how the study size was arrived at	6-7
Quantitative variables	11	Explain how quantitative variables were handled in the analyses. If applicable, describe which groupings were chosen and why	7
Statistical methods	12	(*a*) Describe all statistical methods, including those used to control for confounding	7-8
		(*b*) Describe any methods used to examine subgroups and interactions	7-8
		(*c*) Explain how missing data were addressed	8
		(*d*) If applicable, explain how loss to follow-up was addressed	NA
		(*e*) Describe any sensitivity analyses	NA
**Results**			
Participants	13*	(a) Report numbers of individuals at each stage of study—eg numbers potentially eligible, examined for eligibility, confirmed	9, Suppl

		eligible, included in the study, completing follow-up, and analysed	
		(b) Give reasons for non-participation at each stage	9, Suppl
		(c) Consider use of a flow diagram	Suppl
Descriptive data	14*	(a) Give characteristics of study participants (eg demographic, clinical, social) and information on exposures and potential confounders	9,20
		(b) Indicate number of participants with missing data for each variable of interest	9,20
		(c) Summarise follow-up time (eg, average and total amount)	9
Outcome data	15*	Report numbers of outcome events or summary measures over time	9-11, 21-27
Main results	16	(*a*) Give unadjusted estimates and, if applicable, confounder-adjusted estimates and their precision (eg, 95% confidence interval). Make clear which confounders were adjusted for and why they were included	9-11, 21-27
		(*b*) Report category boundaries when continuous variables were categorized	NA
		(*c*) If relevant, consider translating estimates of relative risk into absolute risk for a meaningful time period	NA
Other analyses	17	Report other analyses done—eg analyses of subgroups and interactions, and sensitivity analyses	9-11
**Discussion**			
Key results	18	Summarise key results with reference to study objectives	11
Limitations	19	Discuss limitations of the study, taking into account sources of potential bias or imprecision. Discuss both direction and magnitude of any potential bias	13-14
Interpretation	20	Give a cautious overall interpretation of results considering objectives, limitations, multiplicity of analyses, results from similar studies, and other relevant evidence	11-14
Generalisability	21	Discuss the generalisability (external validity) of the study results	11-14
**Other information**			
Funding	22	Give the source of funding and the role of the funders for the present study and, if applicable, for the original study on which the present article is based	15

*Give information separately for exposed and unexposed groups.

Note: An Explanation and Elaboration article discusses each checklist item and gives methodological background and published examples of transparent reporting. The STROBE checklist is best used in conjunction with this article

(freely available on the Web sites of PLoS Medicine at http://www.plosmedicine.org/, Annals of Internal Medicine at http://www.annals.org/, and Epidemiology at http://www.epidem.com/). Information on the STROBE Initiative is available at http://www.strobe-statement.org.
